# Effects of biophilic design-based sports facilities on exercise continuation intention: mediating effects of exercise immersion and moderating effect of environmental awareness

**DOI:** 10.3389/fpsyg.2025.1623057

**Published:** 2025-09-08

**Authors:** Kyongmin Lee, Sung-Jun Park

**Affiliations:** ^1^Department of Physical Education, Dong-A University, Busan, Republic of Korea; ^2^Department of Architectural Engineering, Keimyung University, Daegu, Republic of Korea

**Keywords:** biophilic design features of sports facilities, exercise immersion, environmental awareness, exercise continuation intention, mediation analysis, moderation effect

## Abstract

The purpose of this study is to empirically analyze how biophilic design elements in sports facilities influence exercise immersion and the intention to continue exercising. Specifically, the study explores the psychological mechanisms underlying this relationship by testing the mediating effect of exercise immersion and the moderating effect of environmental awareness. A structured survey was administered to 200 physical education majors in Busan and Daegu, South Korea. Correlation analysis, regression analysis, and both mediating and moderating effect analysis were performed to confirm the relationships among the key variables. The findings indicate that biophilic design components positively affect both cognitive and behavioral immersion, and that exercise immersion significantly predicts the intention to continue exercising. Moreover, the psychological/physical effects and indirect natural elements partially mediated the relationship between biophilic design and exercise continuation intention through exercise immersion. Specifically, the mediation analysis revealed that, for psychological/physical effects, the indirect pathway via cognitive immersion accounted for 26.9% of the total effect and via behavioral immersion for 51.1%. For indirect natural elements, the proportions were 20.8% (cognitive immersion) and 25.9% (behavioral immersion). These results indicate substantial, though not exclusive, mediation effects. On the other hand, environmental awareness did not exhibit a statistically significant moderating effect. These findings highlight the psychological mechanisms through which spatial design in sports facilities fosters deeper engagement and sustained participation. The study suggests that strategically incorporating biophilic design elements may enhance user experience and promote long-term exercise participation. This research contributes both theoretically and practically to the development of sustainable sports facilities through an integrative perspective combining sports psychology and environmental design.

## 1 Introduction

In contemporary society, regular physical activity is widely recognized as a critical factor not only in enhancing individual health but also in reducing public healthcare expenditures and promoting overall social welfare. Despite its well-documented benefits, many individuals discontinue exercise participation within a short period of initiating physical activity (Song et al., 2019). This low rate of exercise adherence contributes to an increase in chronic diseases, heightened psychological stress, and widening health disparities (Kim, 2014).

In recent years, increasing attention has been paid to how the built environment—particularly architectural and spatial design—can influence physical activity behavior (Humpel et al., 2004; Titze et al., 2005). Spatial elements such as natural lighting, visual aesthetics, material textures, and environmental cues have been found to affect users' mood, motivation, and perceived comfort, thereby playing a role in exercise initiation and maintenance (Owen et al., 2004; Jung et al., 2024). Environmental psychology suggests that well-designed spaces may not only reduce barriers to exercise but also enhance psychological engagement, potentially improving adherence (Yeh et al., 2016; Marques et al., 2019). In this context, biophilic design offers a promising framework for creating supportive exercise environments.

Rooted in Wilson's Biophilia Hypothesis, which posits that humans possess an innate tendency to connect with nature, biophilic design seeks to integrate natural elements into architectural and spatial environments (Wilson, 1984). Biophilic spaces—characterized by the inclusion of natural light, vegetation, organic materials, landscape views, and nature-inspired forms—have been shown to reduce stress, enhance cognitive function, and promote emotional wellbeing (Choe et al., 2019; Asim et al., 2020). Applications of biophilic design are increasingly evident not only in healthcare (Milliken et al., 2023), educational (Peters and D'penna, 2020), and office settings (Hutson and Hutson, 2023), but also in sports facility design (Tabassum and Park, 2024).

However, empirical research on the structural pathways through which biophilic design in sports facilities influences the psychological responses and continued participation behaviors of exercisers remains limited. In particular, existing studies have largely focused on how spatial design affects static variables such as exercise satisfaction or environmental satisfaction (Livesay and Finn, 2025; Zhang and Dai, 2024; Hwang and Shin, 2021). Few studies have examined the dynamic pathways leading to exercise continuation intention by analyzing the qualitative aspects of psychological experiences during exercise, such as flow.

Furthermore, an individual's level of environmental awareness may shape their spatial experience, highlighting the need to explore whether environmental awareness serves as a moderating variable in the relationship between biophilic design and exercise immersion (Chen, 2017; Xie et al., 2022). Sports facilities are not merely functional spaces but also potential settings for emotional immersion and identity formation, making it essential to consider how users' environmental perceptions vary by individual characteristics.

Environmental awareness is generally conceptualized as a multidimensional construct encompassing cognitive (knowledge and understanding of environmental issues), affective (emotional attachment and empathy toward nature), and behavioral (intention and actions for environmental conservation) dimensions (Schultz, 2001). While this study adopts this theoretical definition, for the purposes of empirical analysis we operationalized environmental awareness as a unidimensional variable that integrates these three dimensions. This approach allows for a comprehensive yet parsimonious measurement of participants' overall environmental perceptions within biophilic sports facilities.

Accordingly, this study sets out to achieve the following objectives. First, it examines the effects of biophilic design components in sports facilities on exercise immersion and the intention to continue exercising. Second, it tests the mediating role of exercise immersion in this relationship. Third, it investigates whether environmental awareness moderates the relationship between biophilic design and exercise immersion. Through this analysis, the study aims to empirically establish an integrated causal framework linking sports environment design, exercise psychology, and sustained exercise participation, thereby contributing to both theoretical understanding and practical implementation.

## 2 Theoretical background and literature review

### 2.1 Biophilic design: definition, framework, and application in sports facilities

Biophilic design is an architectural and environmental design approach grounded in the theory of biophilia, which posits that humans have an innate desire to connect with nature (Kellert et al., 2008). Originally introduced by Edward O. Wilson and later expanded by Kellert, the concept of biophilic design aims to enhance mental and physical wellbeing through the integration of natural light, vegetation, water, fresh air, natural materials, and organic forms within built environments (Tabassum and Park, 2024).

The application of biophilic design in various spatial contexts has been shown to generate multiple benefits, including psychological stability, improved cognitive performance, and enhanced physical health. For instance, a study on biophilic elements in hospital environments reported a reduction in patient stress levels and a shorter recovery period (Zare et al., 2021). Research on biophilic public spaces in urban settings has also demonstrated de-creased stress and enhanced social cohesion among users (Milliken et al., 2023). Similarly, studies conducted in educational environments have found that elements such as natural light, plants, water features, and ventilation contribute to improved cognitive functioning among students (Almusaed et al., 2022).

In recent years, a growing number of studies have reported positive applications of biophilic design in sports-related environments, including fitness centers, indoor gyms, and outdoor exercise spaces. The incorporation of biophilic elements—such as green spaces, natural materials, and visual access to nature—into gyms and fitness facilities has been shown to enhance users' immersion and psychological resilience (Umezinwa et al., 2024). Moreover, biophilic design in sports facilities targeting children and older adults has contributed to the transformation of these spaces into holistic wellbeing environments, where physical activity and mental stability are effectively integrated (Russo and Andreucci, 2023). These findings demonstrate that biophilic design is not only applicable to sports facilities but also holds promise as a foundational strategy for creating sustainable and user-centered physical activity environments.

Table 1 outlines Kellert et al.'s (2008) six categories of biophilic design, including environmental features, natural shapes and forms, natural patterns and processes, light and space, place-based relationships, and evolved human–nature relationships. Each category describes a specific way in which natural elements can be incorporated into built environments to promote psychological stability, sensory stimulation, and emotional attachment.

**Table 1:** Summary of Kellert et al.'s (2008) six categories of biophilic design.

**Category**	**Description**
Environmental features	Incorporates natural elements such as sunlight, plants, water, air, fire, landscapes, and natural materials that users can directly perceive through their senses. These elements are integrated into spaces to promote psychological stability and recovery.
Natural shapes and forms	Uses shapes and forms inspired by nature, such as curves, spirals, and biomorphic patterns derived from plants and animals, to provide visual stability and aesthetic pleasure.
Natural patterns and processes	Reflects temporal and sequential characteristics found in nature such as seasonal changes, life cycles, sensory variety, and ecological flows. These features stimulate a sense of vitality and cognitive stimulation.
Light and space	Utilizes natural views, paintings, reflections, focal points, sense of openness, and depth to create rich visual and emotional spatial experiences.
Place-based relationships	Reflects locality, history, culture, and ecological identity to help users form a sense of belonging and emotional attachment to the space.
Evolved human-nature relationships	Comprises elements that evoke innate emotional responses formed through human evolutionary adaptation to nature, such as balance, complexity, prospect and refuge, and feelings of awe or fascination.

However, subsequent studies have restructured these categories into three integrated dimensions to enhance empirical applicability and design efficiency: direct Experience of Nature, Indirect Experience of Nature, and Experience of Space and Place (Lee and Park, 2023; Kang and Kim, 2024; Kim and Park, 2024).

Drawing on previous studies (Kellert et al., 2008; Kim and Park, 2024; Kim M. J., 2024), this study conceptualizes the characteristics of biophilic design in sports facilities through four sub-dimensions: psychological/physical effects, differentiated experience provision, direct elements of nature, and indirect elements of nature.

Psychological/physical effects refer to the extent to which biophilic design elements promote users' psychological stability and physical recovery within sports facilities. These include benefits such as enhanced concentration, inspiration, creativity, anxiety re-lief, and stress reduction (Kim M. J., 2024). Exposure to natural elements—such as natural light, vegetation, views, and air flow—can facilitate mental clarity and restore vitality (Kellert et al., 2008; Kim and Park, 2024; Kim M. J., 2024).

Differentiated experience provision is defined as the degree to which biophilic design offers a fresh and creative spatial experience distinct from conventional sports facilities (Kim M. J., 2024). This dimension contributes to emotional immersion and subjective satisfaction by stimulating user interest, reinforcing thematic emotional responses, and encouraging a renewed perception of the space (Kim M. J., 2024).

Direct elements of nature involve components that enable users to engage with nature through direct sensory experience, particularly sight and touch (Kellert et al., 2008; Kim and Park, 2024; Kim M. J., 2024). Examples include access to natural light and outdoor views through windows, the presence of indoor plants, ventilation, and physical vegetation (Kellert et al., 2008; Kim and Park, 2024; Kim M. J., 2024). These elements foster relaxation and psychological comfort by activating sensory pathways.

Indirect elements of nature are physically mediated components that symbolically evoke natural settings and promote environmental affinity (Kim and Park, 2024; Kim M. J., 2024). These include visual features such as natural imagery, materials, color schemes, and organic patterns embedded within the space (Kim and Park, 2024; Kim M. J., 2024). Typical examples are wooden finishes, green and brown hues, fractal-patterned structures, and nature-inspired artwork (Kim and Park, 2024; Kim M. J., 2024).

In recent years, a growing number of sports facilities have adopted biophilic design strategies in response to increased awareness of their psychological, physiological, and emotional impacts on users. To identify the spatial characteristics and recurring design intentions across such examples, we analyzed ten biophilic sports facilities across seven countries—Singapore, the United Kingdom, the United States, China, Denmark, Japan, and South Korea—as summarized in Table 2. Each case was categorized based on location, major design elements, biophilic features, and experiential attributes.

**Table 2:** Case studies of biophilic design implementation in contemporary sports facilities.

**No**.	**Facility (Location)**	**Image**	**Biophilic design Elements**	**Key features**
1	Singapore Sports Hub (Singapore)	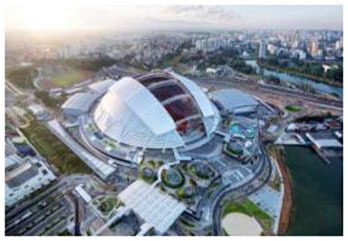 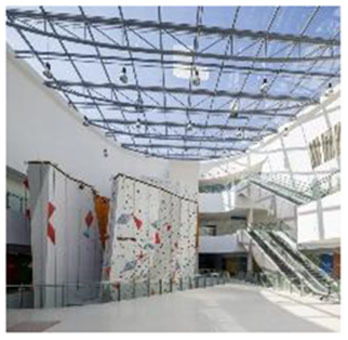	Waterfront view, daylight system, green terrace, open canopy, vegetation, visual-tactile experiences	Integration of retractable roof and natural elements; everyday sports-nature hub aligned with Singapore's Garden City
[15pt] 2	Deep Sea Museum & Sports Center (China)	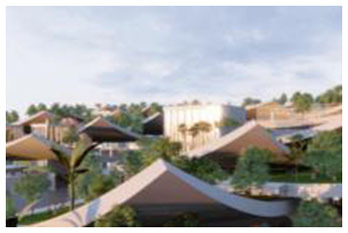 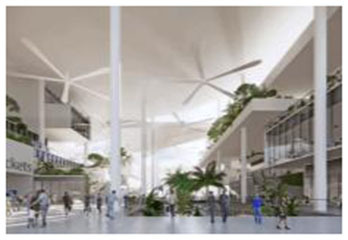	Wave-like roof, landscape integration, daylight control, nature-evoking geometry	Marine-inspired curved design; minimizes heat and solar gain while maximizing connection to outdoor environment
3	Tønder Sports Hall (Denmark)	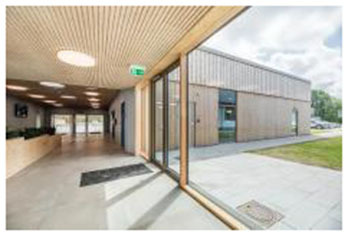 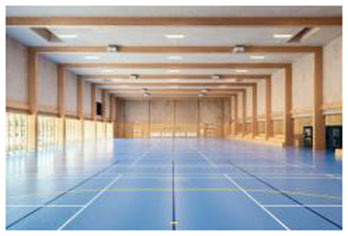	Sustainable cladding, daylight maximization, transparency, wood textures	Thermo wood interior with warm ambiance; strong visual connection via transparent corridors and glazed façades
4	Yoyogi National Gymnasium (Japan)	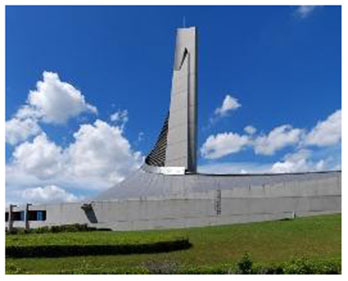 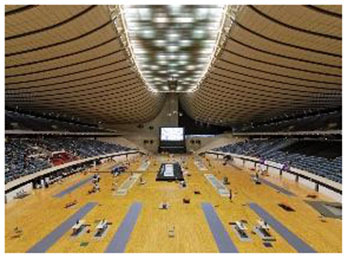	Dynamic roof, natural materials, suspended structure, traditional-meets-organic form	Renovation respecting historical identity; biomorphic design evoking traditional Japanese architecture & landscape
5	Metropolitan Fitness Center (South Korea)	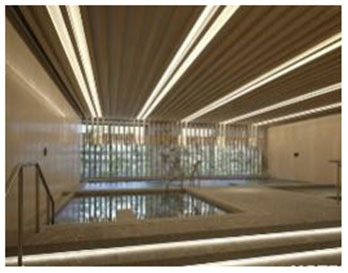 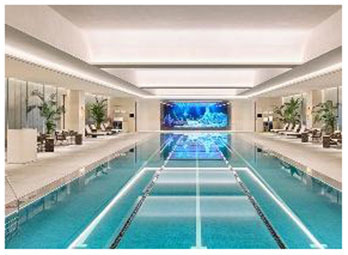	Indoor vegetation, glass curtain wall, daylight system, wooden ceiling, water elements	Restorative spaces via seasonal vegetation and water display; integrated emotional comfort and spatial rhythm
6	Fitness Concord (South Korea)	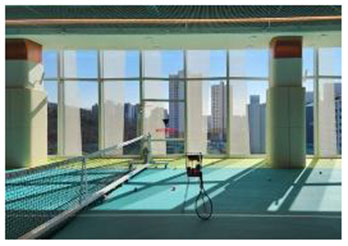 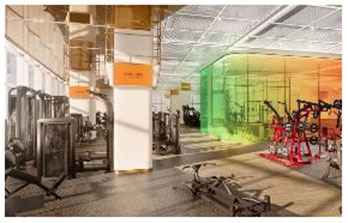	Panoramic glazing, indoor planting, color-light interaction, daylight modulation	Enhanced indoor-outdoor view; emotional stimulation through greenery and lighting

Image Sources

1. Singapore Sports Hub (Singapore) . (https://www.e-architect.com/singapore/singapore-sports-hub), used with permission.

(https://www.e-architect.com/london/london-aquatics-centre), used with permission.

(https://www.theplan.it/eng/architecture/la-garage-at-nike-world-headquarters-in-beaverton-oregon-usa), used with permission.

2. DeepSea Museum & Sports Center (Sanya, China) (https://www.adfwebmagazine.jp/en/design/architectural-design-office-aoe-designed-deep-sea-museum-and-sports-center-in-sanya-china/), used with permission.

3. Tønder Sports Hall (Tønder, Denmark) (https://www.e-architect.com/denmark/tonder-sports-hall-southern-denmark), used with permission.

4. Yoyogi National Gymnasium (Tokyo, Japan) (https://www.archiweb.cz/en/b/olympijske-haly), used with permission.

5. Metropolitan Fitness Center (Seoul, South Korea) (https://seoul.intercontinental.com/grandicparnas/facility/Fitness), used with permission.

6. Fitness Club Concord (Suwon, South Korea) (https://m.starfield.co.kr/suwon/entertainment/concord), used with permission.

First, many facilities prioritized maximizing natural daylight and visual openness to strengthen the visual connection between indoor and outdoor environments. The Tønder Sports Hall in Denmark featured operable window systems and skylights to enable passive lighting, while the Metropolitan Fitness Center in South Korea implemented floor-to-ceiling windows that provided unobstructed views of the surrounding landscape.

Second, the integration of vegetation and water features emerged as a common design strategy to stimulate sensory engagement and support restorative experiences. The Singapore Sports Hub incorporated interior planting and was situated adjacent to a waterfront promenade, allowing for direct and indirect interactions with natural elements. The Metropolitan Fitness Center combined indoor greenery with a reflective water wall to enhance visual and auditory stimulation, while Fitness Concord in Suwon, South Korea, used a combination of plantings and ambient lighting to create an emotionally responsive atmosphere within the indoor environment.

Third, several facilities adopted biomorphic forms and natural geometries to evoke a sense of organic continuity. The Yoyogi National Gymnasium in Japan featured a wave-like, tensile roof that was inspired by traditional Japanese carpentry and the fluid motion of nature. Likewise, the Deep Sea Museum & Sports Center in China incorporated a curved façade designed to simulate the undulating form of ocean waves, effectively reinforcing the thematic connection to nature.

Fourth, the use of natural materials such as wood, stone, and bamboo contributed to a tactile sense of warmth and enhanced the multisensory qualities of the space. The Tønder Sports Hall made extensive use of Thermo wood and exposed timber structures throughout the facility, fostering a warm and natural ambiance. Similarly, the Yoyogi Gymnasium integrated wooden panels to convey both traditional and contemporary aesthetics, further emphasizing biophilic material expression.

Finally, some facilities were designed to support immersive and multifunctional spatial experiences that extended beyond the boundaries of physical activity. The Singapore Sports Hub functioned as an integrated civic, cultural, and athletic complex, combining nature, movement, and community in a cohesive and experiential environment.

### 2.2 Exercise immersion

Flow, as introduced by (Csikszentmihalyi 1975), refers to an optimal psychological state characterized by complete absorption in an activity, a loss of self-consciousness, and intrinsic enjoyment. This state can occur across various domains, including work, arts, gaming, and sports. Exercise immersion is conceptually rooted in the flow framework but is domain-specific, focusing exclusively on physical activity and sports participation. It emphasizes sustained attentional focus, cognitive absorption, and behavioral engagement during exercise (Jackson and Eklund, 2004).

While flow describes a universal psychological phenomenon (Kim Y. et al., 2023), exercise immersion incorporates the unique physical and contextual factors inherent to sports settings, such as bodily movement, spatial interaction, and physiological feedback (Choi et al., 2022). Exercise immersion is typically conceptualized as comprising two dimensions: cognitive immersion and behavioral immersion. Cognitive immersion refers to a mental state involving task-focused concentration, time distortion, and diminished self-awareness (Park et al., 2021a). In contrast, behavioral immersion denotes the manifestation of committed or automated behaviors during task execution (Park et al., 2021a). For example, a study of screen baseball participants found that both cognitive and behavioral immersion positively influenced users' intentions to revisit and recommend the experience (Yu and Lee, 2024), suggesting that immersion may play a critical role in the sustainability and behavioral transition of sports participants.

An increasing number of studies have examined the mediating or moderating role of flow in relation to environmental and psychological factors within sports contexts, such as social presence and self-efficacy. For instance, (Kim et al. 2022) reported that real-time social presence in online fitness environments significantly promoted immersion, which in turn either fully or partially mediated users' intentions to continue participation.

In this study, exercise immersion is defined as the optimal psychological state in which sports participants become deeply absorbed in physical activity to the extent that they lose track of time and self-awareness. It is operationalized as comprising two sub-dimensions: cognitive immersion (e.g., task focus, time distortion) and behavioral immersion (e.g., commitment to performance, automated behavioral responses).

### 2.3 Intention to continue exercise

Intention to continue exercise refers to an individual's sustained commitment to engage in physical activity over time, driven by both intrinsic motivation and established behavioral patterns (Bum and Park, 2015). It encompasses the internal desire to maintain participation for personal satisfaction, health benefits, or enjoyment, as well as the habitual incorporation of exercise into daily life (Ryan et al., 1997).

In previous research, exercise continuation intention has frequently been employed as a dependent variable influenced by diverse psychosocial and environmental factors. Studies have identified several antecedents to exercise continuation intention, including satisfaction of basic psychological needs such as autonomy, competence, and relatedness (Noh et al., 2018); leadership and coaching ability (Jun et al., 2023); the type of feedback provided by instructors (Qichen and Jeong, 2024); quality of educational services (Kim and Nam, 2023); mentoring experiences (Kim, 2019; Seo et al., 2020); and satisfaction with facility environments (Lee et al., 2019). Furthermore, recent research has identified exercise flow as a key mediating variable that plays both direct and indirect roles in predicting the intention to continue exercising (Seo et al., 2020; Lee et al., 2018). These findings suggest that the intention to continue exercise is not merely a reflection of behavioral metrics such as frequency or duration, but a higher-order construct that encompasses psychological acceptance and sustained volitional commitment.

In line with this theoretical context, the present study conceptualizes the intention to continue exercise as the outcome variable and empirically examines how the environ-mental factor of biophilic design influences this intention through the mediating role of exercise flow. Through this investigation, the study seeks to contribute to the field of environmental psychology by clarifying the structural pathways through which environmental design elements shape psychological commitment to sustained physical activity.

This conceptualization is also supported by Self-Determination Theory (SDT), which emphasizes that sustained engagement in physical activity is influenced by the satisfaction of three basic psychological needs—autonomy, competence, and relatedness (Ryan et al., 1997). Biophilic environments may contribute to fulfilling these needs by providing emotionally engaging, self-directed, and competence-enhancing exercise contexts. Therefore, the immersive qualities fostered by biophilic design are not only aesthetically or emotionally valuable but also psychologically motivating in line with SDT.

### 2.4 Environmental awareness

Environmental awareness is a multidimensional concept that encompasses the cognitive, emotional, and behavioral responses that individuals have to the natural and artificial environments surrounding them (Schultz, 2001). It is distinguished from environmental attitudes or environmental values in that it goes beyond simple knowledge or interest in environmental issues and includes the will to practice environmental conservation and emotional bonds (Kim M. S., 2024). In other words, environmental awareness is considered a key element that explains how individuals interpret and experience the environment and what attitudes and behaviors they exhibit accordingly.

The components of environmental awareness are typically classified into three dimensions: cognitive, emotional, and behavioral (Dunlap et al., 2000). The cognitive dimension refers to knowledge, understanding, and information related to environmental or ecological issues. The emotional dimension encompasses feelings such as attachment, empathy, and a sense of belonging toward natural or eco-friendly spaces. The behavioral dimension involves the intent and actual practice of environmentally responsible behavior, which reflects one's environmental ethics and sense of responsibility (Kollmuss and Agyeman, 2002).

In this study, environmental awareness was measured with four items that collectively reflect all three dimensions: one cognitive item (“I believe small individual efforts can contribute to environmental improvement”), one affective item (“I believe environmental issues deserve attention even if they do not directly affect me”), and two behavioral items (“I consider environmental issues when selecting sports-related products and services” and “I am interested in environmental issues and actively practice environmental protection for future generations”). Although these items represent different dimensions, prior research in sports and leisure contexts has shown that these dimensions are often highly intercorrelated and can be validly combined into a single latent factor to streamline measurement and reduce respondent burden (e.g., Niu et al., 2024; Santos-Pastor et al., 2022).

This unidimensional operationalization is also theoretically supported by Ajzen's (1991) Theory of Planned Behavior, which posits that attitudes, affect, and behavioral intentions are interrelated components of a broader evaluative orientation. Accordingly, in this study, environmental awareness was modeled as a single factor representing an overall pro-environmental orientation relevant to sports facility contexts.

Recent studies have demonstrated that environmental awareness positively influences not only quality of life, emotional stability, and wellbeing, but also the continuity of exercise and leisure activities (Pretty et al., 2005). Research suggests that in biophilic environments—spaces incorporating natural elements—positive environmental perceptions are closely associated with spatial satisfaction, psychological immersion, and behavioral persistence (Hung and Chang, 2021; Ortegón-Cortázar and Royo-Vela, 2019).

From this perspective, the possibility that environmental awareness can affect users' psychological experience and behavioral responses in living spaces such as sports facilities has been raised. In fact, research results have been reported that environmental awareness of users of eco-friendly sports facilities has a positive effect on exercise participation attitude and immersion (Kim J. et al., 2023; Hou et al., 2023). This suggests that sports facilities with biophilic design can play an important role in the psychological experience and behavioral changes of users beyond mere physical space.

Accordingly, this study establishes environmental awareness as a moderating variable that affects exercise immersion in sports facilities based on biophilic design and empirically investigate its role.

## 3 Research hypothesis

### 3.1 Effects of biophilic design on exercise immersion

For immersion to occur, appropriate environmental stimulation and emotional stability must be provided together (Csikszentmihalyi, 1975), and in particular, the physical and psychological characteristics of the exercise environment can exert a critical influence on this immersive experience (Kim et al., 2024; Park et al., 2021b). In this context, biophilic design has gained attention as an effective spatial strategy that promotes immersion. Grounded in the biophilia hypothesis—proposing that humans have an innate desire to connect with nature—biophilic de-sign integrates natural elements such as daylight, vegetation, airflow, natural materials, and visual access to nature into the built environment. These components are known to reduce user stress and enhance concentration and resilience (Tabassum and Park, 2024; Kellert et al., 2008).

Indeed, several empirical studies have reported increased immersion among exercise participants in sports facilities incorporating biophilic design. (Artha et al. 2022) found that the use of plants, natural lighting, and material finishes in fitness centers significantly enhanced users' immersion and psychological recovery. Similarly, (Tjia et al. 2024) observed improvements in both immersion and participation sustainability when biophilic design was applied to indoor sports facilities for older adults. Additionally, (Almusaed et al. 2022) demonstrated that educational environments incorporating natural light and plant elements enhanced students' concentration and cognitive functioning—suggesting potential applicability to exercise spaces as well.

These previous studies suggest that biophilic components in sports facilities create favorable sensory, emotional, and environmental conditions for immersion, ultimately strengthening individuals' intention to continue exercising through the mediating influence of immersion.

Accordingly, the following hypotheses are proposed:

H1. Components of biophilic design-based sports facilities have a positive (+) effect on exercise immersion.

H1-1. Psychological/physical effects have a positive (+) effect on exercise immersion.

H1-2. Provision differentiated experiences has a positive (+) effect on exercise immersion.

H1-3. Direct elements of nature have a positive (+) effect on exercise immersion.

H1-4. Indirect elements of nature have a positive (+) effect on exercise immersion.

### 3.2 The effect of exercise immersion on continuation intention

It is widely recognized that the intention to continue exercising is influenced by both basic psychological needs—such as autonomy, competence, and relatedness, as proposed in Self-Determination Theory—and internal experiential factors, including immersion, satisfaction, and enjoyment (Noh et al., 2018; Seo et al., 2020). Among these factors, exercise immersion is a state of psychological immersion that occurs during exercise, and refers to a phenomenon in which an individual forgets the passage of time and experiences the best emotional state while deeply immersed in exercise (Csikszentmihalyi, 1975). Immersion enhances intrinsic motivation and evokes positive emotions, thereby serving as a critical factor in promoting sustained exercise participation by increasing satisfaction with the activity (Choi et al., 2022; Kim Y. et al., 2023).

Prior research on exercise immersion has found that both dimensions (i.e., cognitive immersion and behavioral immersion) significantly influence exercise continuation intention. For example, (Yu and Lee 2024) reported that higher levels of immersion were associated with stronger intentions to re-engage in exercise and to recommend it to others. These findings suggest that immersion functions as more than a transient psychological state; it is a key predictor of emotional attachment and long-term behavioral commitment to exercise.

This relationship has been repeatedly confirmed in empirical studies. For example, (Choi et al. 2022) reported in a study targeting Taekwondo gymnastics participants that organizational identification enhances immersion and that immersion has a positive effect on the intention to continue to participate in exercise, and Kim Y. et al. (2023) demonstrated that fitness appusers experience immersion through elements such as challenge and re-ward, and as a result, their intention to continue to use the app is strengthened. In addition, (Oh and Kim 2023) empirically analyzed how immersion among smartwatch users positively affected both emotional engagement and sustained exercise behavior. Together, these studies underscore immersion as a key pathway variable in explaining exercise continuation intention. These studies underscore immersion as a key pathway variable in explaining exercise continuation intention. Based on these findings, this study proposes the following hypothesis:

H2. Exercise immersion has a positive effect on the intention to continue exercising.

### 3.3 Effects of biophilic design components on the intention to continue exercising

Previous studies have shown that biophilic design environments contribute not only to enhancing emotional responses, stress regulation, and cognitive functioning (Hung and Chang, 2021), but also to improving the quality of physical activity participation, including exercise (Peters and D'penna, 2020). In particular, when natural elements were incorporated into sports facilities, increases were reported in users' psychological stability, immersion, and intentions for reuse and continued participation (Tabassum and Park, 2024; Umezinwa et al., 2024; Xiao et al., 2025).

Building on this evidence, the present study seeks to examine how the subcomponents of biophilic design in sports facilities—namely psychological/physical effects, pro-vision of differentiated experiences, direct elements of nature, and indirect elements of nature—individually influence the intention to continue exercising. These elements are hypothesized to enhance behavioral commitment through mechanisms such as emotional stability, satisfaction with a novel exercise environment, and a sense of connectedness to nature.

Accordingly, the following hypotheses are proposed:

H3. The composition factors of biophilic design-based sports facilities have a positive effect on the intention to continue exercising.

H3-1. Psychological/physical effects have a positive effect on the intention to continue exercising.

H3-2. Provision of differentiated experiences has a positive effect on the intention to continue exercising.

H3-3. Direct elements of nature have a positive effect on the intention to continue exercising.

H3-4. Indirect elements of nature have a positive effect on the intention to continue exercising.

### 3.4 The mediating role of exercise immersion

Continued participation in exercise is not solely achieved by providing physical space; rather, users' psychological immersion in the environment, along with the experience of positive emotions and intrinsic motivation, serves as a critical mediating pathway. In particular, exercise immersion functions as a key intermediary variable through which the characteristics of the exercise environment influence sustained behavioral engagement (Seo et al., 2020; Lee et al., 2018).

Immersion is an optimal psychological state characterized by deep concentration, loss of self-awareness, and time distortion (Csikszentmihalyi, 1975). This state enables individuals to become fully absorbed in the activity, leading to emotional satisfaction and a sense of achievement. Exercise immersion fosters positive affect and enjoyment, thereby serving as a crucial psychological mechanism that enhances the intention to continue participating in exercise (Choi et al., 2022).

Biophilic design-based sports facilities integrate natural elements—such as daylight, vegetation, natural materials, colors, and scenic views—to create environments that promote psychological stability and immersion (Tabassum and Park, 2024; Kellert et al., 2008). In a study by (Umezinwa et al. 2024), participants demonstrated significantly greater immersion in biophilic fitness spaces, which in turn was associated with higher levels of exercise satisfaction and a stronger intention to maintain participation.

Similarly, (Kim et al. 2022) empirically identified a mediating structure in which sensory stimuli (e.g., color, interactive elements) within the exercise environment induced immersion, which subsequently enhanced the intention to continue using the facility. These findings suggest that environmental design elements may influence behavioral outcomes indirectly through the psychological mechanism of immersion, rather than through direct effects alone.

In summary, a structural pathway exists in which the physical and psychological stimulation provided by biophilic design features induces exercise immersion, which then reinforces intrinsic motivation and the behavioral intention to continue exercising. Accordingly, the following hypothesis is proposed:

H4. Exercise immersion mediates the relationship between biophilic design-based sports facility components and the intention to continue exercising.

### 3.5 The moderating role of environmental awareness

Even within the same biophilic design environment, individuals' immersion responses may differ depending on their level of environmental awareness. Those with high sensitivity to nature and strong eco-friendly values tend to respond more positively to natural elements—such as plants, daylight, and landscape views—experience greater emotional stability, and are more likely to enter a state of psychological immersion (Chen, 2017). Conversely, individuals with low environmental concern may show reduced sensitivity to the same spatial stimuli and exhibit weaker immersive engagement (Chen, 2017).

Empirical studies support this notion. For instance, Kim M. S. (2024) found that individuals with higher environmental awareness rated eco-friendly sports facilities more positively and reported greater immersion and intention to continue using the space. Similarly, (Pretty et al. 2005) reported that stronger emotional immersion and psychological recovery occurred among individuals with high environmental attachment in nature-friendly environments.

These findings suggest that environmental awareness may function as a moderating variable in the relationship between biophilic design elements and exercise immersion. In other words, the impact of biophilic environments on immersion may differ depending on individuals' levels of environmental awareness. This makes environmental awareness a meaningful explanatory factor in understanding the interaction between environmental design stimuli and psychological responses.

Accordingly, the following moderating hypothesis is proposed:

H5. Environmental awareness moderates the relationship between biophilic design-based sports facility components and exercise immersion.

**Figure 1:**
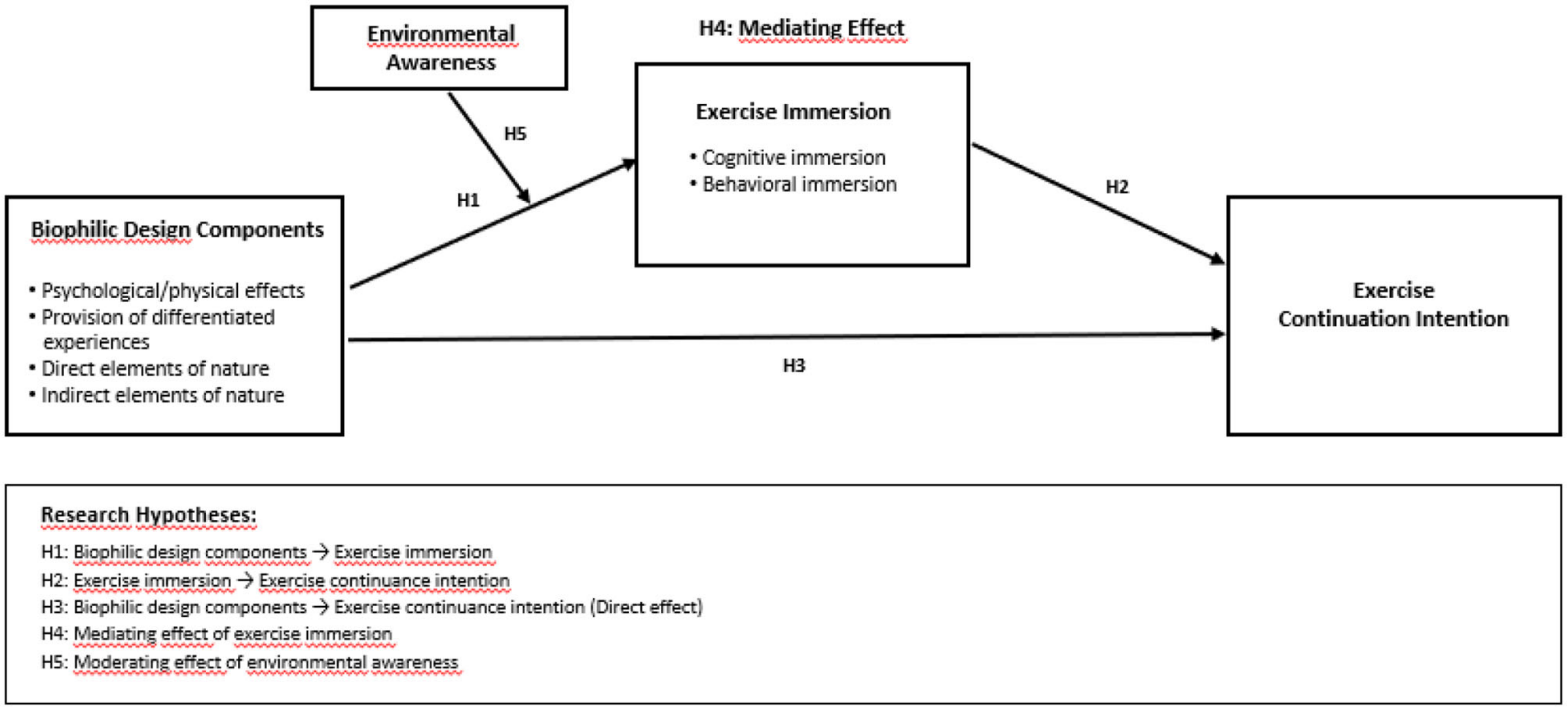
Research model.

Figure 1 illustrates the proposed research model of this study. The model posits that biophilic design components in sports facilities—psychological/physical effects, provision of differentiated experiences, direct elements of nature, and indirect elements of nature—positively influence exercise continuation intention. This relationship is mediated by exercise immersion (cognitive and behavioral immersion), while environmental awareness is included as a moderating variable that may strengthen or weaken the effect of biophilic design on exercise immersion.

## 4 Research method

### 4.1 Research subjects

Participants in the study were undergraduate students majoring in physical education from the Busan and Daegu regions who had prior experience with sports facilities incorporating biophilic design elements, including fitness centers, community gyms, and public sports complexes. The sample was obtained using a convenience sampling method. Before participating, individuals were provided with a consent form that explained the study's purpose, ensured anonymity, and emphasized voluntary participation. Only those who provided informed consent were included in the survey.

Data were collected through both an online survey platform (e.g., Google Forms) and direct responses obtained during on-site visits. Of the 210 questionnaires distributed, 204 were deemed valid and included in the final analysis after excluding those with missing or insincere responses. Table 3 displays the demographic characteristics of the sample.

**Table 3:** General characteristics of subjects.

**Category**		**Frequency**	**Percentage**
Gender	Male	110	53.9
	Female	94	46.1
Year	Freshman	43	21.1
	Sophomore	87	42.6
	Junior	30	14.7
	Senior	44	21.6
Age	Under 21	40	19.6
	21 to 22	80	39.2
	23 to 24	34	16.7
	25 and above	50	24.5

### 4.2 Measurement tool

The measurement instrument employed in this study was a structured questionnaire, which included items related to biophilic design-based sports facility characteristics, exercise immersion, exercise continuation intention, environmental awareness, and participants' general demographics. Except for demographic items, all constructs were assessed using a 5-point Likert scale.

To verify the construct validity of the instrument, an exploratory factor analysis (EFA) was conducted. Reliability was assessed using Cronbach's alpha (α) coefficients. The scale measuring biophilic design-based sports facility characteristics was adapted and refined from a questionnaire originally developed by Kim M. J. (2024), which itself was based on prior studies. The revised scale consisted of 16 items grouped into four subdimensions: psychological/physical effects, provision of differentiated experiences, direct elements of nature, and indirect elements of nature.

Psychological/physical effects were measured with four items assessing the extent to which facility users experienced (a) greater mental clarity and enhanced concentration, (b) increased creativity and inspiration, (c) improved memory and cognitive performance, and (d) reduced anxiety and stress.

Provision of differentiated experiences was measured with four items evaluating perceptions of (a) novelty, (b) thematic uniqueness, (c) a fresh and creative spatial atmosphere, and (d) increased interest in participating in programs due to the biophilic design.

Direct elements of nature were measured with four items capturing user experiences of (a) natural light, (b) views of natural scenery through windows, (c) presence of indoor plants or greenery, and (d) ventilation or fresh airflow.

Indirect elements of nature were measured with four items assessing the presence of (a) nature-inspired imagery or artwork, (b) natural materials such as wood or stone, (c) color schemes resembling natural hues (green, brown, gray), and (d) biomorphic or fractal patterns reflecting natural forms.

Table 4 presents the exploratory factor analysis and reliability results for the biophilic design scale used in this study. The four subdimensions—psychological/physical effects, provision of differentiated experiences, direct elements of nature, and indirect elements of nature—explained a total variance of 71.103%. Cronbach's α values ranged from 0.834 to 0.889, indicating high internal consistency. These results confirm that the scale reliably measures the intended constructs within the context of sports facilities.

**Table 4:** Validation of a questionnaire on biophilic design characteristics in sports facilities: A factor and reliability approach (*N* = 204).

**Item**	**Loadings**	**α**
**Psychological/physical effects**		0.889
I experienced greater mental clarity and enhanced concentration when using a sports facility incorporating biophilic design elements.	0.871	
I felt inspired and experienced enhanced creativity while using a sports facility incorporating biophilic design elements.	0.826	
I experienced clearer thinking and improved memory while using a sports facility with biophilic design elements.	0.814	
I felt less anxious and less stressed while using a sports facility with biophilic design elements.	0.773	
**Providing a differentiated experience**		0.834
I perceive sports facilities with biophilic design elements as offering a differentiated and unique experience.	0.799	
I feel a sense of novelty when using a sports facility with biophilic design elements.	0.780	
I feel as if I am experiencing a new theme when using a sports facility with biophilic design elements.	0.746	
I became more interested in participating in exercise programs at sports facilities due to the application of biophilic design elements.	0.687	
**Direct elements of nature**		0.853
I feel that using a biophilic sports facility reflects my identity as someone ahead of the eco-friendly trend.	0.867	
I feel psychological comfort when I see the natural scenery through the window in a sports facility.	0.843	
I feel psychological comfort when I receive natural light through the window in a sports facility.	0.749	
I feel psychologically comfortable when I see vines and potted plants inside a sports facility.	0.662	
**Indirect elements of nature**		0.847
I feel psychological comfort when I see picture frames with natural images in sports facilities.	0.820	
I feel psychological comfort when I touch natural materials such as wood, stone, and soil in sports facilities.	0.810	
I feel psychological comfort when I see colors that resemble natural colors (brown, green, gray) in sports facilities.	0.801	
I feel psychological comfort when I see fractals with natural shapes (leaves, snowflake shapes, etc.) in sports facilities.	0.762	

The exercise immersion questionnaire was adapted from items used in previous studies, with modifications based on the instrument employed in (Chun 2020). The sub-factors of exercise immersion consisted of six items for cognitive immersion and six items for behavioral immersion. Results from the factor analysis indicated that the questionnaire explained 76.586% of the variance. The reliability of the questionnaire showed high internal consistency, with Cronbach's alpha values ranging from 0.917 to 0.953 (see Table 5).

**Table 5:** Validation of a questionnaire on exercise immersion: a factor and reliability approach (*N* = 204).

**Item**	**Loadings**	**α**
**Cognitive immersion**		0.953
I am proud of participating in this exercise.	0.881	
I plan to continue doing this exercise in the future.	0.873	
I always look forward to the time I spend doing this exercise.	0.863	
This exercise is a very important part of my life.	0.852	
Thinking about this exercise makes me happy.	0.850	
I feel a great sense of happiness from this exercise.	0.848	
**Behavioral immersion**		0.917
If I had more time, I would like to do this exercise more often.	0.871	
This exercise is my most preferred activity during my leisure time.	0.841	
I make an effort to obtain information about the techniques or methods related to this exercise.	0.809	
If there are articles or TV broadcasts about the exercise I do, I tend to prioritize watching or reading them.	0.795	
I often imagine myself performing this exercise impressively.	0.773	
I feel like I am deeply immersed in this exercise.	0.721	

The items measuring exercise continuation intention were adapted from the studies of (Kang 2017) and (Heo 2021), based on the questionnaire developed by (Lee 2022). This con-struct was measured using four items under a single factor:

(1) “I will continue to participate in the exercise I am currently engaged in,”

(2) “I believe the exercise I am participating in is important in my life,”

(3) “I want to continue participating in the exercise in the future if time allows,” and

(4) “I will recommend the exercise I am doing to people around me.”

The reliability coefficient (Cronbach's α) for this scale was 0.918.

Items measuring environmental awareness were modified and supplemented based on the questionnaires used in the studies by (Kim and Choi 2024), as well as (Seo and Lee 2025). This construct also consisted of four items representing a single factor:

(1) “I believe environmental issues deserve attention even if they do not directly affect me.”

(2) “I consider environmental issues when selecting sports-related products and services.”

(3) “I believe small individual efforts can contribute to environmental improvement.”

(4) “I am interested in environmental issues and actively practice environmental protection for future generations.”

The reliability coefficient (Cronbach's α) for this scale was 0.810.

### 4.3 Data analysis

Initially, the construct validity of each measurement variable was established through exploratory factor analysis (EFA), while the internal reliability of the scale was assessed utilizing Cronbach's α coefficient. Subsequently, correlation analysis among the variables and multiple regression analysis were executed, along with the regression analysis as posited by (Baron and Kenny 1986) to ascertain the mediating effect of exercise immersion and the moderating effect of environmental awareness. The data analysis was carried out employing the SPSS 30.0 software program.

## 5 Results

### 5.1 Correlation analysis

The findings from the correlation analysis pertaining to the principal variables of this investigation are delineated in Table 6. All study variables were positively and significantly correlated. The strongest association was found between behavioral immersion and exercise continuation intention (*r* = 0.641, *p* < 0.01), followed by cognitive immersion and exercise continuation intention (*r* = 0.575, *p* < 0.01). Environmental awareness exhibited moderate positive correlations with both cognitive immersion (*r* = 0.411, *p* < 0.01) and behavioral immersion (*r* = 0.451, *p* < 0.01). All correlation coefficients were below 0.9, indicating no multicollinearity issues.

**Table 6:** Correlation between the characteristics of sports facilities based on biophilic design, exercise immersion, environmental awareness, and exercise continuation intention (*N* = 204).

**Variable**	**1**	**2**	**3**	**4**	**5**	**6**	**7**	**8**
Effect	1							
Experience	0.554^**^	1						
Direct factor	0.368^**^	0.419^**^	1					
Indirect factor	0.205^**^	0.346^**^	0.432^**^	1				
Cognitive immersion	0.357^**^	0.402^**^	0.399^**^	0.381^**^	1			
Behavioral immersion	0.357^**^	0.430^**^	0.363^**^	0.369^**^	0.568^**^	1		
Environmental awareness	0.231^**^	0.307^**^	0.399^**^	0.358^**^	0.411^**^	0.451^**^	1	
Exercise continuation intention	0.421^**^	0.474^**^	0.465^**^	0.515^**^	0.575^**^	0.641^**^	0.565^**^	1

^**^p < 0.01.

### 5.2 Hypothesis verification

The mediating effect of exercise immersion in the relationship between biophilic design-based sports facility characteristics and exercise continuation intention was examined using the three-step regression analysis method proposed by (Baron and Kenny 1986).

In the first regression model (see Table 7), where the characteristics of biophilic design-based sports facilities were entered as independent variables and exercise immersion as the dependent variable, the subdimensions—psychological/physical effect (β = 0.154, *p* < 0.05), differentiated experience (β = 0.167, *p* < 0.05), direct nature elements (β = 0.181, *p* < 0.05), and indirect nature elements (β = 0.214, *p* < 0.01)—were found to have significant positive effects on cognitive immersion. In addition, effect (β = 0.227, *p* < 0.01) and indirect elements (β = 0.207, *p* < 0.01) had significant positive effects on behavioral immersion. These results partially supported Hypothesis H1, with all sub-hypotheses (H1-1 to H1-4) confirmed for cognitive immersion, and H1-1 and H1-4 supported for behavioral immersion.

**Table 7:** Verification of the mediating effect of exercise immersion in the relationship between biophilic design-based sports facility characteristics and exercise continuation intention (*N* = 204).

**DV**	**IV**	***B***	***S.E*.**	**β**	***T***	***F***	***R^2^***
Cognitive immersion	Effect	0.152	0.073	0.154	2.092^*^	18.923^***^	0.276
	Experience	0.186	0.086	0.167	2.164^*^		
	Direct	0.193	0.077	0.181	2.514^*^		
	Indirect	0.209	0.067	0.214	3.126^**^		
Behavioral immersion	Effect	0.265	0.090	0.227	2.934^**^	18.449^***^	0.271
	Experience	0.147	0.077	0.142	1.922		
	Direct	0.142	0.081	0.127	1.756		
	Indirect	0.212	0.070	0.207	3.014^**^		
Exercise continuation intention	Effect	0.177	0.062	0.188	2.854^*^	35.988^***^	0.420
	Experience	0.190	0.073	0.179	2.600^*^		
	Direct	0.178	0.065	0.175	2.718^**^		
	Indirect	0.315	0.057	0.338	5.532^***^		
Exercise continuation intention	Effect	0.129	0.058	0.138	2.214^*^	39.222^***^	0.498
	Experience	0.132	0.069	0.125	1.915		
	Direct	0.117	0.062	0.116	1.897		
	Indirect	0.249	0.054	0.268	4.591^***^		
	Cognitive immersion	0.311	0.056	0.328	5.539^***^		
	Effect	0.120	0.055	0.128	2.181^*^		
	Experience	0.088	0.066	0.083	1.341	43.631^***^	0.524
	Direct	0.123	0.058	0.121	2.120^*^		
	Indirect	0.233	0.051	0.251	4.545^***^		
	Behavioral immersion	0.383	0.051	0.423	7.579^***^		

^*^p < 0.05, ^**^p < 0.01, ^***^p < 0.001.

In the second regression model (see Table 7), in which the biophilic design-based facility characteristics were the independent variables and exercise continuation intention was the dependent variable, all four subdimensions showed significant positive effects: effect (β = 0.188, *p* < 0.01), experience (β = 0.179, *p* < 0.05), direct (β = 0.175, *p* < 0.01), and indirect (β = 0.338, *p* < 0.001). Accordingly, Hypothesis H3 and all its sub-hypotheses (H3-1 to H3-4) were supported.

In the third regression model (see Table 7), both the biophilic design-based facility characteristics and exercise immersion were entered as independent variables, with exercise continuation intention as the dependent variable. Cognitive immersion was found to significantly predict exercise continuation intention (β = 0.328, *p* < 0.001). The regression coefficients for effect (from β = 0.188 to β = 0.138) and indirect (from β = 0.338 to β = 0.268) decreased compared to step 2, indicating a partial mediating effect of cognitive immersion. Even in the third model, both effect (β = 0.138, *p* < 0.05) and indirect (β = 0.268, *p* < 0.001) remained significant, further supporting partial mediation.

Similarly, behavioral immersion significantly influenced exercise continuation intention (β = 0.423, p < 0.001). The coefficients for effect (from β = 0.188 to β = 0.128) and indirect (from β = 0.338 to β = 0.251) were reduced, indicating that behavioral immersion also partially mediated the relationship. The continued significance of effect (β = 0.128, *p* < 0.05) and indirect (β = 0.251, *p* < 0.001) confirms this mediation.

Therefore, both Hypotheses H2 and H4 were supported. These findings suggest that exercise immersion serves as a key mechanism in enhancing individuals' intention to continue exercising. Moreover, among the biophilic design characteristics, psychological/physical effects and indirect natural elements exert an indirect influence on exercise continuation intention through immersion. Specifically, the bootstrapping results indicated that the proportion of the total effect accounted for by the indirect effect was 26.9% for psychological effects, 51.1% for physical effects, and 25.9% for indirect natural elements, respectively, indicating partial mediation in all three cases.

The three-step regression analysis method proposed by (Baron and Kenny 1986) was employed to examine the moderating effect of environmental awareness on the relationship between biophilic design-based sports facility characteristics and cognitive immersion.

In the first regression model (see Table 8), where biophilic design-based sports facility characteristics were entered as independent variables, the subdimensions—psychological/physical effect (β = 0.154, *p* < 0.05), differentiated experience (β = 0.167, *p* < 0.05), direct nature elements (β = 0.181, *p* < 0.05), and indirect nature elements (β = 0.214, *p* < 0.01)—were found to significantly and positively predict cognitive immersion.

**Table 8:** Verification of the moderating effect of environmental awareness on the relationship between biophilic design-based sports facility features and cognitive immersion (*N* = 204).

**Step**	**Variables**	***B***	***S.E*.**	**α**	***t***	***F***	***R^2^***
1	Effect	0.152	0.073	0.154	2.092^*^	18.923^***^	0.276
	Experience	0.186	0.086	0.167	2.164^*^		
	Direct	0.193	0.077	0.181	2.514^*^		
	Indirect	0.209	0.067	0.214	3.126^**^		
2	Effect	0.145	0.071	0.147	2.036^*^	18.208^***^	0.315
	Experience	0.158	0.084	0.141	1.874		
	Direct	0.133	0.077	0.124	1.724		
	Indirect	0.165	0.067	0.168	2.473^*^		
	Environment	0.226	0.067	0.224	3.375^**^		
3	Effect	0.115	0.074	0.116	1.547	10.354^***^	0.324
	Experience	0.167	0.085	0.150	1.959		
	Direct	0.124	0.079	0.116	1.566		
	Indirect	0.169	0.069	0.173	2.448		
	Environment	0.244	0.070	0.242	3.491		
	Effect x Environment	0.039	0.053	0.066	0.741		
	Experience x Environment	−0.057	0.055	−0.096	−1.037		
	Direct x Environment	0.017	0.051	0.027	0.333		
	Indirect x Environment	−0.045	0.054	−0.071	−0.820		

^*^p < 0.05, ^**^p < 0.01, ^***^p < 0.001.

In the second regression model (see Table 8), which included both the biophilic design-based facility characteristics and environmental awareness as predictors, effect (β = 0.147, *p* < 0.05), indirect elements (β = 0.168, *p* < 0.05), and environmental awareness (β = 0.224, *p* < 0.01) were positively associated with cognitive immersion.

In the third regression model (see Table 8), which included interaction terms between the subdimensions of biophilic design characteristics and environmental awareness, none of the interaction effects reached statistical significance.

These findings indicate that environmental awareness does not moderate the relationship between biophilic design-based sports facility characteristics and cognitive immersion.

In the first-step regression model (see Table 9), in which biophilic design-based sports facility characteristics were entered as independent variables, the subdimensions effect (β = 0.227, *p* < 0.01) and indirect elements (β = 0.207, *p* < 0.01) were found to have a significant positive effect on behavioral immersion.

**Table 9:** Verification of the moderating effect of environmental awareness on the relationship between biophilic design-based sports facility characteristics and behavioral immersion (*N* = 204).

**Step**	**Variables**	***B***	***S.E*.**	**α**	***T***	***F***	***R* ^2^**
1	Effect	0.265	0.090	0.227	2.934^**^	18.449^***^	0.271
	Experience	0.147	0.077	0.142	1.922		
	Direct	0.142	0.081	0.127	1.756		
	Indirect	0.212	0.070	0.207	3.014^**^		
2	Effect	0.228	0.087	0.195	2.616^*^	19.902^***^	0.334
	Experience	0.137	0.073	0.132	1.866		
	Direct	0.061	0.080	0.055	0.771		
	Indirect	0.153	0.069	0.149	2.219^*^		
	Environment	0.302	0.069	0.286	4.362^***^		
3	Effect	0.235	0.076	0.201	2.681^**^	11.755^***^	0.353
	Experience	0.132	0.088	0.127	1.727		
	Direct	0.044	0.081	0.039	0.537		
	Indirect	0.119	0.071	0.116	1.678		
	Environment	0.310	0.072	0.293	4.318^***^		
	Effect x Environment	−0.051	0.055	−0.082	−0.930		
	Experience x Environment	−0.067	0.057	−0.107	−1.177		
	Direct x Environment	0.026	0.052	0.038	0.488		
	Indirect x Environment	0.022	0.056	0.033	0.395		

^*^p < 0.05, ^**^p < 0.01, ^***^p < 0.001.

In the second-step model (see Table 9), which included both biophilic design-based facility characteristics and environmental awareness as a moderator variable, the effect (β = 0.195, *p* < 0.05), indirect elements (β = 0.149, *p* < 0.05), and environmental awareness (β = 0.206, *p* < 0.001) were each positively associated with behavioral immersion.

In the third-step regression model (see Table 9), which incorporated interaction terms between biophilic design-based characteristics and environmental awareness, none of the interaction variables had a statistically significant effect on cognitive engagement.

These findings indicate that environmental awareness does not moderate the relationship between biophilic design-based sports facility characteristics and behavioral engagement. Therefore, Hypothesis H5 was rejected, as no significant interaction effect was observed between environmental awareness and exercise immersion in the form of behavioral engagement.

## 6 Discussion and conclusion

### 6.1 Theoretical implications

This study contributes to the growing body of research at the intersection of environmental psychology, biophilic design, and physical activity behavior by empirically analyzing the effects of biophilic design elements on exercise immersion and exercise continuation intention. The following theoretical implications can be drawn from the major findings.

First, the components of biophilic design-based sports facilities were found to have a significant positive impact on both cognitive and behavioral immersion. This aligns with prior research suggesting that nature-integrated environments enhance emotional stability, attentional focus, and mental restoration (Almusaed et al., 2022; Artha et al., 2022; Tjia et al., 2024). The identification of indirect natural elements (e.g., natural materials, visual stimuli) and psychological/physical effects as key predictors of immersion reinforces the theoretical understanding that immersive user experiences in built environments are shaped by multi-sensory, restorative cues. This may be because indirect elements can be flexibly integrated into indoor sports facilities where direct access to outdoor natural environments is limited. Such elements provide consistent and visually accessible cues that evoke a sense of nature without the constraints of location, weather, or maintenance costs associated with direct natural features. Additionally, prior research has shown that symbolic representations of nature can trigger restorative responses comparable to those elicited by direct exposure, especially in urban or enclosed environments (Kellert et al., 2008; Hung and Chang, 2021). Therefore, the strong impact of indirect natural elements observed in this study underscores their practical and scalable value in promoting immersive and sustained exercise participation.

Second, both cognitive and behavioral immersion significantly predicted users' intention to continue exercise, providing empirical support for immersion theory (Csikszentmihalyi, 1975; Choi et al., 2022; Kim Y. et al., 2023) in the context of physical activity settings. This finding expands the conceptualization of exercise continuation intention by highlighting immersion as a central psychological mechanism, suggesting that long-term engagement in exercise is influenced not merely by behavioral repetition but by deeply engaging emotional and cognitive experiences.

Third, exercise immersion was shown to play a partial mediating role in the relationship between biophilic design characteristics and exercise continuation intention. This advances theoretical models of environmental influence on behavior by revealing the indirect psychological pathway through which spatial stimuli affect user outcomes. The evidence that environmental features impact behavioral intentions via immersion underscores the importance of internal psychological states in mediating the environment-behavior link.

Fourth, environmental awareness was hypothesized as a moderating variable but was found not to significantly alter the relationship between biophilic design characteristics and exercise immersion. This finding implies that psychological responses to biophilic environments may operate independently of individual differences in environmental consciousness, thus lending further support to the foundational claim of biophilia theory—that humans possess an innate, universal affinity for natural elements. The lack of moderation suggests that biophilic effects on immersion may be broadly generalizable across populations regardless of their ecological awareness or pro-environmental attitudes.

### 6.2 Theoretical and practical, and policy implications

This study structurally clarified the influence of environmental design factors—specifically biophilic design—on exercise psychology and behavioral intention, offering the following theoretical, practical, and policy-related implications.

From a theoretical perspective, this study contributes to academic discourse by empirically examining the effects of biophilic design through an integrated lens encompassing sports psychology, environmental psychology, and sports facility design. While previous research has often focused on facility satisfaction or isolated emotional responses, the present study advances theory by structurally elucidating the psychological pathway from immersion to behavioral intention. The demonstration of exercise immersion as a mediating mechanism reinforces the significance of internal psychological experiences in predicting sustained exercise participation. These findings suggest the potential for developing an emotion–environment integrated model, which complements existing behavioral theories such as Self-Determination Theory (SDT) and the Theory of Planned Behavior (TPB). Furthermore, the finding that environmental awareness did not moderate the relationship between biophilic design and immersion suggests that affective responses to nature-based environments may be broadly universal, thus providing a fresh theoretical lens for understanding individual differences in environmental design responsiveness.

From a practical standpoint, several implications arise for the design, management, and operation of sports facilities. First, facility planners and designers should prioritize not only functional efficiency but also psychological engagement and emotional comfort through spatial strategies. The use of natural light, eco-friendly materials, indoor greenery, and nature-inspired color schemes can effectively enhance users' immersive experiences at a relatively low cost, especially during renovations. Second, in the context of marketing and operations, cultivating a nature-integrated atmosphere can serve as a key driver for increasing user dwell time and repeat visits, positioning biophilic design as a central element in customer experience management. It is therefore essential to view environmental design not merely as an aesthetic enhancement, but as a strategic tool that influences user behavior and emotional engagement.

From a policy perspective, the findings provide a rationale for integrating biophilic design into public health and urban development strategies. Local governments and public sector facility operators are encouraged to adopt biophilic principles in the planning and renovation of sports facilities, particularly within eco-friendly urban policy frameworks. Such initiatives can simultaneously address goals related to environmental sustainability and community health promotion by fostering environments that support both psychological wellbeing and active lifestyles. Policymakers should consider incentivizing the implementation of biophilic elements through building codes, funding schemes, or certification systems, thereby promoting widespread adoption of psychologically beneficial design across public sports infrastructure.

### 6.3 Limitations of the study and future tasks

While this study empirically investigated the effects of biophilic design-based sports facilities on exercise immersion and exercise continuation intention—along with the mediating and moderating mechanisms involved—it is not without limitations. The following considerations should be taken into account when interpreting the findings, and they point to fruitful directions for future research.

First, the study sample consisted exclusively of college students majoring in physical education from specific regions in South Korea (Busan and Daegu). This narrow demographic scope limits the generalizability of the results, as the findings may not reflect the perceptions or behaviors of individuals from different age groups, educational backgrounds, or lifestyle contexts. Future studies should expand the sample to include a more diverse population in terms of age, occupation, geographic region, and exercise experience, thereby enhancing the external validity of the results.

In addition, the sample size was relatively modest for the scope of variables examined. While it was sufficient to conduct the planned statistical analyses (Hair et al., 2019), a larger sample could yield more precise estimates and enhance the robustness of the results. Future studies should consider increasing the sample size by expanding recruitment to multiple institutions, incorporating online and offline data collection channels, and targeting users with varying exercise frequencies and facility usage patterns.

Second, another limitation concerns the coverage of different exercise frequencies and types among participants. Although all respondents had prior experience with biophilic sports facilities, the sample did not explicitly ensure proportional representation across various exercise frequencies (e.g., less than once per week, 1–2 times, 3–4 times, 5 or more times per week) or activity types (strength training, team sports, mixed activities). This may limit the applicability of the findings to specific subgroups of exercise participants. Future research should aim to stratify samples to include diverse exercise behaviors for more comprehensive generalization.

Third, this study relied on self-reported measures, which are susceptible to measurement error and biases such as social desirability and recall bias. Observational assessments by trained evaluators could complement self-reported data to capture non-verbal indicators of immersion and engagement. Triangulating multiple data sources (e.g., surveys, sensor data, and observations) would not only reduce bias but also provide richer, multi-dimensional insights into participants' exercise experiences and environmental perceptions.

Fourth, the use of a cross-sectional design precludes definitive causal inferences. Although significant relationships were identified among biophilic design characteristics, immersion, and exercise continuation intention, the temporal sequence of these variables remains unclear. Future research should adopt longitudinal or experimental designs to more rigorously examine the causal pathways between evolving immersion experiences and long-term behavioral commitment to exercise.

Fifth, environmental awareness was measured as a unidimensional construct, which may have limited the study's ability to capture the full range of cognitive, emotional, and behavioral dimensions associated with environmental perception. To better assess the moderating role of environmental awareness, future research should consider developing and applying multidimensional measures that reflect individuals' values, knowledge, attitudes, and behaviors regarding environmental issues.

Sixth, another limitation is the absence of detailed information regarding participants' specific exercise habits or usage patterns of biophilic sports facilities. Future studies should incorporate such variables to better contextualize behavioral responses.

Finally, this study did not account for other psychological and environmental variables that may influence exercise continuation intention, such as spatial satisfaction, self-efficacy, emotional wellbeing, or spatial identity. These factors may interact with immersion or biophilic design in complex ways. Therefore, future studies should aim to construct a more integrated and multi-layered theoretical model that incorporates these additional constructs to provide a more comprehensive understanding of the psychological mechanisms underpinning exercise continuation intention.

## Data Availability

The original contributions presented in the study are included in the article/supplementary material, further inquiries can be directed to the corresponding author.
